# Cost of mate choice: Changing patterns of global age disparity in marriage and their consequences to women’s health including maternal mortality and menopause

**DOI:** 10.1177/17455057241264687

**Published:** 2024-07-27

**Authors:** Mindy Pru, C Michelle Brown, Rama S Singh

**Affiliations:** Department of Biology, McMaster University, Hamilton, ON, Canada

**Keywords:** age disparity, marriage, mate choice, maternal mortality, menopause

## Abstract

**Background:**

Consistent across cultures and throughout time is the male preference for younger females. Given its prevalence, the mate choice theory proposes that age–disparate relationships may have contributed to the evolution of maternal mortality and menopause.

**Objectives:**

The objective is to document evidence for age disparity in marriage from past and present populations and evaluate their relevance to maternal mortality and menopause.

**Design:**

Cross-sectional data were collected from various regions and time points, ranging from the Roman era to the current decade.

**Methods:**

To analyze both the age disparity in marriage and age at marriage, data were collected from Ancestry.ca for Quebec, Massachusetts, India, South Africa, and England and Wales. Additional data were taken from the United Nations as a more recent and comprehensive source. To analyze the relationships between age disparity in marriage and different social factors, data on gross domestic product, maternal mortality rates, fertility, primary school enrollment, child marriage rates, and percentage of women in the total labor force were collected from the World Health Organization, World Bank, and United Nations International Children’s Emergency Fund.

**Results:**

The results showed that males were significantly older than females at first marriage in all populations and time frames sampled, supporting the assumption underlying the mate choice theory. Maternal mortality rates were strongly associated with age–disparate relationships, increasing by 275 per 100,000 live births for each additional year in the age disparity.

**Conclusion:**

The results from this study provide support for the assumption underlying the mate choice theory of maternal mortality and menopause.

## Plain language summary

Changing patterns of global age disparity in marriage provide support for the mate choice theory of menopause, raising the possibility of a gradual shift in delayed reproduction and delayed menopause. Living things have inherent capacity to survive and reproduce until they die, with one exception: humans. Women go through menopause while men remain fertile. Among the many explanations offered for the origin of menopause, grandmother hypothesis is the leading one. Being too old to reproduce, it is argued, grandmothers stop reproducing and make up for the loss of fitness through helping (grand mothering) their grand offspring. There are two problems with this theory: first, grand mothering and menopause need not be connected, and second, grandmothers cannot be simultaneously too old to reproduce and not too old to be able to gather resources to make up for their loss of fitness. We proposed a mate choice theory of menopause which posits that human mating system is non-random, that is, males have preference for younger females, depriving older females from reproduction and allowing deleterious fertility mutations to accumulate giving rise to menopause. Male preference for younger females is consistent across cultures and dominates all social relations affecting mate choice including monogamy, serial monogamy, widowers remarrying, polygamy, harem, and others. In this study, we wanted to test if male preference for younger females has been widespread through time. We collected data on age disparity in marriage from past and present populations, from Roman era to the current decade, and evaluated their relevance to the origin of maternal mortality and menopause. The results showed that males were significantly older than females at first marriage in all populations and time frames sampled, supporting the assumption underlying the mate choice theory of menopause. Maternal mortality rates were strongly associated with age–disparate relationships, increasing by 275 per 100,000 live births for each additional year in the age disparity. Through repeated cycles of widowers marring younger women, maternal mortality would have functioned as a reinforcer of the origin of menopause.

## Introduction

Among many cultures, there is a prevailing preference for age–disparate relationships involving an older male and a younger female.^1–3^ An analysis of whole-genome data and a mutation model by Wang et al.,^3^ has estimated that in the past 250,000 years, the age disparity between couples was 7.5 years. Child marriages often exhibit an extreme case of age–disparate relationships.^4^ Currently, United Nations International Children’s Emergency Fund (UNICEF) estimates girls below the age of 18 are married almost five times more than boys around the world, and girls below 15 are married more than eight times more.^4^ South Asia and sub-Saharan Africa have the highest rates of child marriage with half of the world’s child brides being in South Asia; India alone contains a third of the global total.^4^ Evidently, many child marriages, and consequently large age disparities between couples, occur in developing nations.^4^ Within-country analyses also show that couples of lower socioeconomic status (SES) and education are more likely to have a greater spousal age gap than couples of higher SES.^5–7^ Recent international efforts have pushed these rates to decline as historical age disparities, and rates of child marriage were even higher.^8^ Such efforts include increases in educational attainment, declines in arranged marriages, and overall changing laws and cultural norms.^8^ While marriage laws prohibiting the union of individuals below a certain age should reduce early marriage, evidence from previous studies suggests it to be less effective than intended, likely due to poor enforcement and monitoring of these laws.^8,9^

With many relationships consisting of an older male and a younger female, raises the question—*Why?* From a resource-based, evolutionary perspective, it was hypothesized that the sexes were faced with different adaptive challenges, such as selecting a partner able to provide or a partner of high reproductive value, which led to the evolution of such relationships.^10,11^ In a large-scale study done by Buss,^12^ females were found to value “good financial prospects” in a potential partner more strongly than males. Conversely, males were found to value “good looks” more strongly than females.^12^ Societal, non-adaptive factors may also have reinforced the attraction to age–disparate relationships. The influence of religion, cultural norms, or societal pressures, for example, can be a driving factor toward the preference.

**Figure 1.**
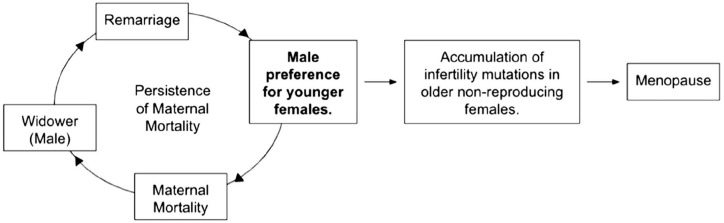
The preference for younger women contributes to the persistence of maternal mortality and the evolution of menopause.

Considering its prevalence, it has been questioned whether there are consequences of age–disparate relationships to women’s health. Two recent studies have explored whether this preference may have contributed to the continued persistence of *maternal mortality* and *menopause*.^13,14^ The World Health Organization (WHO) defines *maternal mortality* as “female deaths from any cause related to or aggravated by pregnancy or its management.”^15^ *Menopause* is defined by the WHO as “the end of monthly menstruation due to loss of ovarian follicular function.”^16^ Theoretically, maternal mortality and menopause should be selected against in a population, but by prioritizing mating with younger females, maternal mortality and menopause may no longer be under negative selection.^13,14^ The mate choice theory proposes that this preference may be contributory toward maternal mortality because females below 15 years are at higher risk of adverse pregnancy outcomes, young females often lack support during pregnancy, and the effects are more severe on female fitness and especially aggravated by polygyny (Figure 1).^13,17–22^ In regard to menopause, the mate choice theory proposes the preference may have resulted in older, non-reproducing women to accumulate infertility mutations responsible for menopause (Figure 1).^14^ Infertility mutations would be rendered neutral in older women as they are no longer reproducing.^14^

The evolution of maternal mortality and menopause is perplexing, but the mate choice theory may provide some clarity. As such, the primary objective of this study was to assess the assumption that males prefer younger females, in the mate choice theory by documenting the age disparity in marriage from past and present populations. We hypothesize that there is and has been an age disparity between couples across cultures. With this study, we hope to help bridge the gap between mate choice, maternal mortality, and menopause by providing evidence for the assumption underlying the theory.

## Methods

The Strengthening the Reporting of Observational Studies in Epidemiology (STROBE) guidelines were followed when preparing this article.^23^ A detailed list of the abbreviations and acronyms used in this article is shown in Table 1. While remarriage is common and relevant to the mate choice theory, our study primarily focused on the age at first marriage to assess the assumption of age-disparate relationships.^24^ Details of the data used, including the list of populations and sources used, are shown in Table 2. Raw data with individual ages from our sample using Ancestry.ca are available on request from authors.

**Table 1.** List of abbreviations used and their definition.

Abbreviation	Definition
AICc	Akaike information criterion
ANOVA	Analysis of variance
CI	Confidence interval
DHARMa	Residual Diagnostics for Hierarchical (Multi-Level/Mixed) Regression Models
GDP	Gross domestic product
GLMM	Generalized linear mixed model
MAD	Median absolute deviation
MMR	Maternal mortality rate
SES	Socioeconomic status
SI	Supplemental Information
STROBE	Strengthening the Reporting of Observational Studies in Epidemiology
UN	United Nations
UNICEF	United Nations International Children’s Emergency Fund
WHO	World Health Organization

**Table 2.** Details for all data used.

Data	Sample size (*N*)	Source		Used in
Quebec	Nm = 605 Nf = 641	Ancestry.ca	Quebec, Canada, Vital, and Church Records (Drouin Collection), 1621–1968	Figure 2
Massachusetts, United States	Nm = 685 Nf = 714	Ancestry.ca	Massachusetts, United States, Town, and Vital Records, 1620–1988	Figure 2
India	Nm = 219 Nf = 241	Ancestry.ca	India, Select Marriages, 1792–1948	Figure 2
South Africa	Nm = 247 Nf = 267	Ancestry.ca	South Africa, Dutch Reformed Church Registers, 1660–1970	Figure 2
England and Wales	Nm = 328 Nf = 342	Ancestry.ca	England and Wales Marriages, 1538–1988	Figure 2
Age disparity at first marriage	N_regions_ = 78	UN	World Marriage Data 2019	Figure 3
Roman	Nm = 168 Nf = 324	*The Age of Roman Girls at Marriage*	Original Source: *Age at Marriage and at Death in the Roman Empire* by Harkness.^24^ Funeral inscriptions from the Corpus Inscriptionum Latinarum were encrypted and recorded by Harkness if the age at marriage could be determined.	Figure 4
Polygyny	Monogamy: Nm = 106 Nf = 106 2 wife: Nm = 99 Nf = 198 3+ wife: Nm = 41 Nf = 132	*Pioneers and Prominent Men of Utah*	Genealogies and Biographies, 1847–1868	Figure 5
Age disparity at first marriage	N_regions_ = 99	UN	World Marriage Patterns 2000	Figures 6, 7, and 8
GDP (per capita)	*N* = 99	World Bank	GDP per capita (current United States Dollar, 2022)	Figures 6 and 8
MMR	*N* = 88	WHO	Trends in Maternal Mortality: 1990–2008	Figure 6
Fertility	*N* = 97	World Bank	Fertility rate, total (births per woman)	Figure 6
Child marriage rate	*N* = 58	UNICEF	Percentage of women aged 20 to 24 years who were first married or in union before age 15 or 18	Figure 7
Women in total labor force	*N* = 93	World Bank	Labor force, female (percentage of total labor force)	Figure 7
Primary school enrollment	*N* = 69	World Bank	Gross enrollment ratio, primary, gender parity index	Figure 7

Nm: number of males; Nf: number of females; UN: United Nations; GDP: gross domestic product; MMR: maternal mortality rate; WHO: World Health Organization; UNICEF: United Nations International Children’s Emergency Fund.

## Comparisons of age at marriage and age disparity

### Data sets of populations

#### Ancestry.ca

The following locations were documented to analyze the age disparity in marriage through time: Quebec (1670–1939), Massachusetts (1720–1989), England and Wales (1850–1970), South Africa (1840–1940), and India (1850–1930).^25–29^ These locations were selected because they had more data available compared with other collections on Ancestry.ca. The purpose of using a diversity in locations was to explore the age disparity in marriage in various parts of the world over time. From Ancestry.ca, an individual’s age at marriage and year of marriage were collected for men and women. Age disparity was then calculated for each region by subtracting the age of men by the age of women at marriage. Hence, a positive age disparity would indicate men being older than women in relationships and vice versa.

#### The United Nations (UN)

To incorporate more diversity and mitigate selection bias, data were also collected from the UN World Marriage Data, which includes the marital status, age, and sex for populations in 159 countries or territories around the world from 1960–2019.^30^ Only relatively recent data were available from the UN data set so we created two separate models to allow for the unique perspective of historic data from Ancestry.ca, while having the bonus of more diverse data from the UN. Data on the age disparity in marriage from the UN (1990–1998) were also used to assess its relationship with other social factors.^31^

#### Roman

A frequency table on the age of men and women at marriage was taken from “The Age of Roman Girls at Marriage” to explore how current estimates of the age disparity compare to estimates from the Roman times.^32^ An exact time frame for the inscriptions was not provided. Funeral inscriptions from the Corpus Inscriptionum Latinarum were encrypted and recorded by Harkness^32^ if the age at marriage could be determined. Any values listed as “Not counted in number” in the article were not included.^32^

#### Polygyny

To assess how mate choice functions in polygynous relationships, data were collected from “Pioneers and Prominent Men of Utah,*”* a genealogy and biography of the Latter-Day Saint pioneers who came to Utah between 1847 and 1868, collected by Frank Esshom.^33^ Individuals’ year at birth and marriage were collected and organized by sex, wife number (e.g. first wife and second wife), and thus family system (e.g. one wife, two wives, and three wives). The data collected represent those born between 1774 and 1885.^33^ Polygynous males were determined based on whether they had a child with a female while also married to another female. Otherwise, it was defined as serial monogamy and not incorporated. The age at marriage was calculated through the year of marriage and the year of birth.

### Analysis

#### Ancestry.ca

Within this data set, 239 outliers were identified and removed from the data set by using the robust *z*-score method from the *performance* package in *R*.^34,35^ This algorithm computes the median absolute deviation (MAD) and removes any values outside 1.959 MADs from the median. The aim of removing outliers was to reduce the impact of widowers and/or significantly older partners remarrying later in life.^24,36^ Finally, we mean centered and standard deviation scaled the ages of each individual and the years of each marriage. Using the data from Ancestry.ca, males’ and females’ age at marriage was compared using a generalized linear mixed model (GLMM) using the package *glmmTMB* in *R*.^34,37^ This model, henceforth called model 1, aimed to investigate whether males were more likely to be older at the age of marriage, and by how much. The first iteration of this model included age as the effect, sex (male or female) as a fixed effect predictor, and random effects of the intercepts of each year and each location. This adjusts the model for the variance in ages between locations and differences in ages that may be found for each year. Once a basic starting model was created, further models with added complexity were added (see Supplemental Information (SI): Model comparisons for write out of each model). Ancestry Model 2 and Ancestry Model 3 had the same fixed effects as model 1, but model 2 added “location” as a random effect, and model 3 added both “year” and “location” as separate random effects. Ancestry Model 4 had the same random effects as model 3 but included year and the interaction of sex and year as a fixed effect. Ancestry Model 5 was similar to model 4, but another fixed effect term was added, location, as well as the interactions with both sex and year. Due to the complexity of having consecutive years’ worth of data from the same location, model 6 was created the same as model 5 with an added Ornstein–Uhlenbeck covariance structure to allow for the inconsistent time periods between the collected data.^38^ Finally, model 7 was the same as model 6 but with an added dispersion parameter of sex, location, and the interaction between sex and location. The seven models were assessed by their log likelihood density and small sample corrected Akaike information criterion (AICc) to determine that model 7 represented the variation in data most accurately (see SI: Model comparisons).

Using the Residual Diagnostics for Hierarchical (Multi-Level/Mixed) Regression Models *(DHARMa)* package, the dispersion, quantiles, distribution, outliers, and autocorrelation of the model were diagnosed with simulated residuals from model 7.^34,39^ These tests found significant outliers (*p* = 0.048) and quantiles that did not match the hypothetical expected values (*p* < 0.01). We determined that the discrepancy between our model and the theoretical model for each of these tests was small (46 outliers at both margins with *N* = 4289; parametric coefficients of −0.019, −0.074, and −0.029 for the 0.25, 0.5, and 0.75 quantiles, respectively) and the robustness of our data set was large enough that we deemed it appropriate to continue on with model 7 (*N* = 4289).

#### UN

The average age of marriage for each sex in a year in one country/territory was collected and used in a GLMM using *glmmTMB*.^34,37^ The ages and years included were standard deviation scaled and mean centered. Then, six models were created. UN Model 1 included the prediction of age of marriage by the sex of the individuals. UN Model 2 included the same fixed effects, but random variation by country/territory was added as a random effect. UN Model 3 was the same as UN Model 2, but included another random effect, year. UN Model 4 introduced an additional fixed effect, year of data collection, and its interaction with the other fixed effect, sex. UN Model 5 included the same fixed effects as UN Model 4, but with an added autoregression covariance structure for the relationship between successive years for each country. Finally, UN Model 6 included sex as a dispersion factor with the same fixed and random effects as UN Model 5, except for the country/territory random effect which caused the model not to converge, so it had to be removed. When comparing the models, UN Model 6 had the lowest AICc and highest log likelihood density, so it was selected as the best fit for the data (see SI: Model comparisons).

To assess the fit of the model, the *DHARMa* package was used, once again, to test the dispersion, quantiles, distribution, outliers, and autoregression of simulated residuals from the model.^34,39^ The dispersion, distribution, and outlier tests were all insignificant.^38^ The quantile test showed significant differences between the theoretical and observed values, and the one-step autoregression test showed significant autoregression. The amount of discrepancy between the theoretical and observed value was very small for these two tests, so although they were significant, they were determined to be acceptable.

#### Roman

Using *glmmTMB*, a linear model was created to predict age using parameters of sex, religion, and the interaction between sex and religion.^34,37^ In observing the data, clear discrepancies in variance between males and females were noted, so a dispersion parameter by sex was added to the model. Simulated residuals were created from the model and dispersion, outliers, uniformity, and quantiles were all assessed using the *DHARMa* package.^34,39^ There were slightly more outliers than expected; otherwise, the tests were insignificant. An analysis of variance (ANOVA) was used to assess the differences between each religious group and each sex, and Tukey’s honest significant difference was implemented to evaluate the specific differences between each sex, religion, and comparisons between one sex from both religions (Table 5).

#### Polygyny

Using the robust *z*-score method from the package *performance*, 17 outliers were removed.^34,35^ A preliminary simple generalized linear model was created predicting the age at first marriage by the sex of the individual, marriage type (i.e. polygynous or monogamous), and the interaction between the two. A second model added the birth year of the individual as a fixed effect and the interaction between birth year and sex. Polygyny Model 2 also had two added random effects, birth year and marriage type. A third polygyny model was built off Polygyny Model 2, with the total number of wives in the relationship, and marriage order (i.e. if the individual was their partner’s first marriage and second marriage), as well as both of the interactions of these with sex. Polygyny Model 3 also added a covariance matrix term to the birth year random effect and separated these by sex. Finally, the fourth model included the same fixed effects as Polygyny Model 3, but the marriage type random effect was removed, and the number of wives in the relationship and sex was added as dispersion parameters. All models were created using the package *glmmTMB*.^34,37^ Comparing the AICc of each model demonstrated the fourth model as the best representation of the data (see SI: Model comparisons). A series of model diagnostic tests were conducted using the *DHARMa* package to assess the dispersion, quantiles, outliers, distribution, autocorrelation, and homogeneity of sex, marriage type, and number of wives.^34,39^ There were some minor discrepancies in the quantiles test that were determined acceptable.

## Potential correlates with age disparity

To examine potential correlates with age disparity, estimates of gross domestic product (GDP) per capita, maternal mortality rate (MMR), and fertility were collected from various countries across all continents, except Antarctica (for a complete list, see SI).^31,35,40–42^ Primary school enrollment, child marriage rates, and the percentage of women in the total labor force were also collected from various countries.^31,43–45^ The factors assessed were selected based on data availability and what has been proposed to influence the age disparity in marriage. Data were time point and country-specific, such that each data point represents a country’s age disparity in marriage and the associated factor at the same time (Figures 7 and 8). Country estimates range from 1990 to 1998. Estimates were all time matched.

### Analysis

Each predictor variable was scaled by its standard deviation and mean centered. Next, two GLMMs were created using the package *glmmTMB*.^34,37^ Social Correlate Model 1 predicted age disparity in marriage using log-transformed GDP per capita and MMRs as fixed effects. The second model added fertility as another fixed effect and the continent each country belongs to as a random effect. In addition, continent was added as a dispersion factor. In comparing between the models, Social Correlate Model 2 was determined to best fit the data (see SI: Model comparisons). In assessing the model fit, simulated residuals were created using the *DHARMa* package.^34,39^ Dispersions, quantiles, zero inflation, distribution, outliers, and autocorrelation were assessed, and none of the tests showed significant differences between the estimated and observed data sets. Primary school enrollment, child marriage rates, and the percentage of women in the total labor force were also collected but because there were fewer entries available for these factors, compared with GDP, MMR, and fertility rates, they were not included in the model.^31,43–45^ Instead, their correlations to age disparity in marriage were assessed using Microsoft Excel.^46^ The relationship between GDP and MMR was also assessed in the same manner.

## Results

The main objective of this study was to evaluate and explore the age disparity in relationships. We will now discuss the significant findings from the regression analyses conducted on each data set as outlined above. Results were concluded as statistically significant if *p* < 0.05.

### Age disparity in marriage

#### Ancestry model

**Figure 2.**
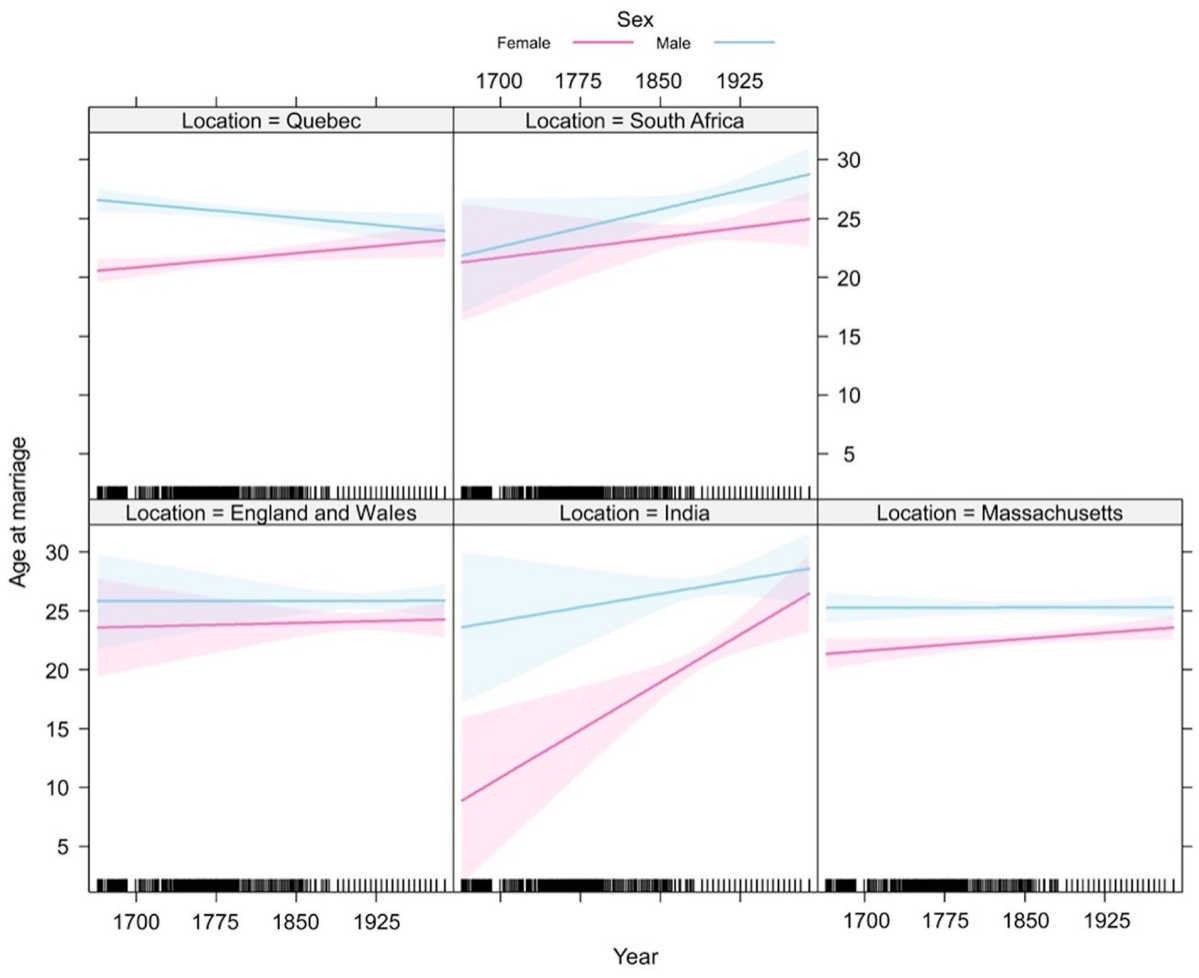
Male (blue) and female (pink) ages at marriage in Quebec Canada, South Africa, England and Wales, India, and Massachusetts, USA from 1670 to 1989 as calculated by the Ancestry model. Shaded regions represent 95% confidence intervals.

The main objective of Figure 2 was to explore how the age at marriage has evolved throughout time. It was found that males were significantly older than females by an average of 3 years for all locations analyzed (*p* = 1.04 × 10^−3^; see SI: Ancestry model; Figure 2). Across all locations, there was no significant change in age of marriage over time, except for India, where the age of marriage increased by 1 year every 19 years (*p* < 0.01); specifically, with females increasing in age 2.75 times faster than males (*p* = 0.04; Figure 2). In addition, India had a significantly younger age at marriage than the global average age by 5 years (*p* < 0.01), with males in India being 6 years older than females (*p* < 0.01). The locations that showed significant differences between their mean age of marriage were England and Wales with India, Massachusetts, and Quebec, and South Africa with India, Massachusetts, and Quebec (Table 3).

**Table 3.** Tukey’s honest significant difference between ages of marriage in each location from Ancestry.ca.

Location	Difference	Lower CI	Upper CI	*p*	Significance
India versus England and Wales	−0.95	−1.68	−0.21	0.00	**
Massachusetts versus England and Wales	−0.74	−1.31	−0.18	0.00	**
Quebec versus England and Wales	−0.76	−1.34	−0.18	0.00	**
South Africa versus England and Wales	0.32	−0.39	1.03	0.74	
Massachusetts versus India	0.20	−0.45	0.85	0.91	
Quebec versus India	0.19	−0.47	0.85	0.94	
South Africa versus India	1.27	0.49	2.04	0.00	***
Quebec versus Massachusetts	−0.01	−0.49	0.46	1.00	
South Africa versus Massachusetts	1.06	0.44	1.69	0.00	***
South Africa versus Quebec	1.08	0.44	1.71	0.00	***

CI: confidence interval.

Significance: **p < 0.01; ***p < 0.001.

#### UN model

**Figure 3.**
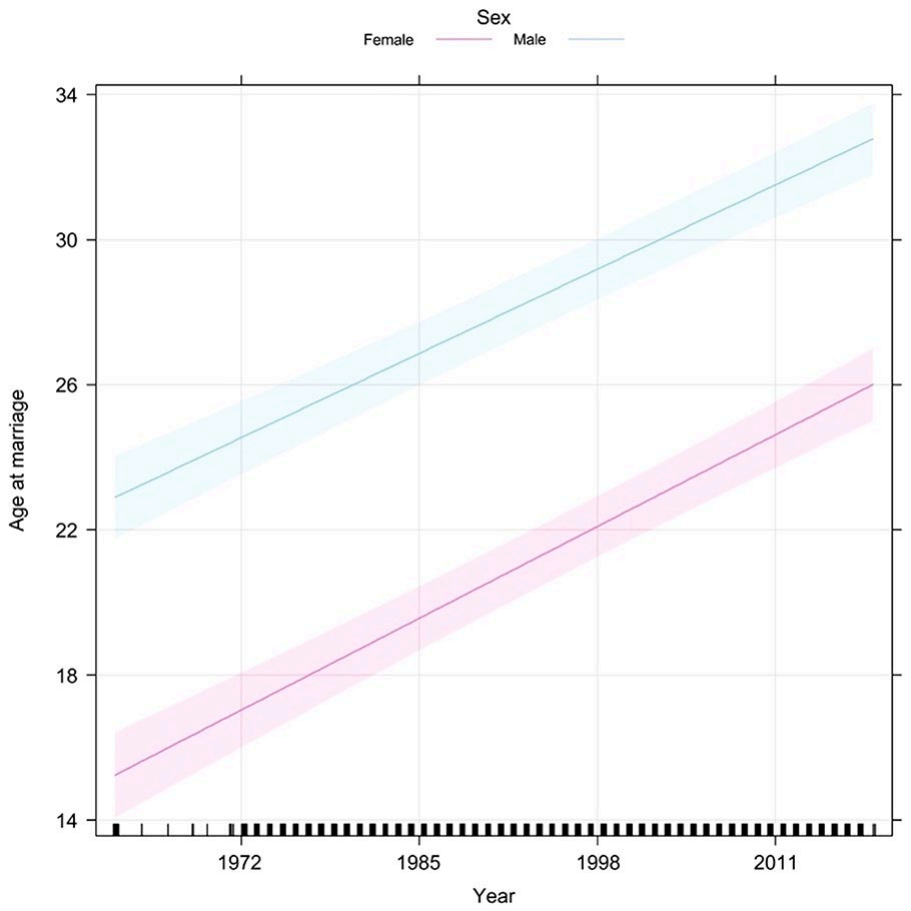
Age of marriage for males (blue) and females (pink) for 78 United Nations-recognized countries and territories around the world between the years 1960 and 2019 as calculated by the United Nations model. Shaded regions represent 95% confidence intervals.

Figure 3 was intended to demonstrate how universal age–disparate relationships are through the global age at marriage. Males were seen to be significantly older than females by 4 years (*p* < 0.01; Figure 3). Over the time the data were recorded, the age of marriage increased by 1 year every 11 years. The male rate of change was similar to the female rate during this period (Table 4).

**Table 4.** Coefficients and significance of model parameters for United Nations model predicting age of marriage.

Parameter	Estimate	Standard error	*z* value	*p*	Significance
Intercept	−0.49	0.05	−9.11	0.00	***
SexMale	0.89	0.01	86.63	0.00	***
Year	0.32	0.02	15.44	0.00	***
SexMale: Year	−0.03	0.01	−2.54	0.01	*

Significance: *p < 0.05; ***p < 0.001.

#### Roman model

**Figure 4.**
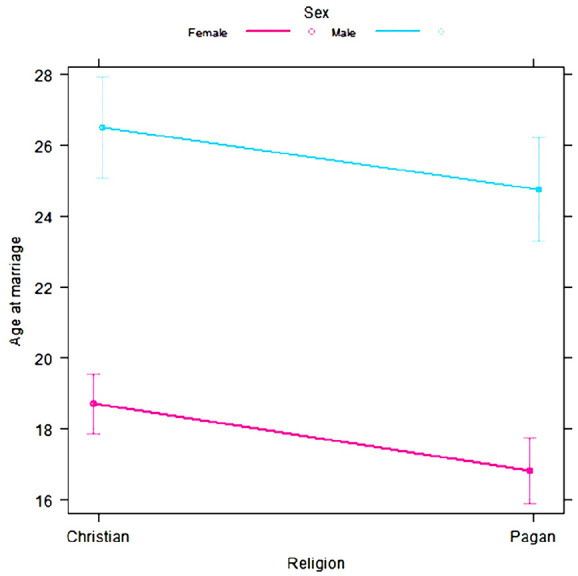
The age of marriage for males (blue) and females (pink) in Christians and Pagans in Ancient Rome as predicted by the Roman model. Error bars represent 95% confidence intervals.

The aim of Figure 4 was to explore the age disparity in marriage in more historical times. Roman couples had an average age disparity of 8 years, with males being older than females (*p* < 0.01; Figure 4). Pagan couples were found to be 2 years younger than their Christian counterparts (*p* < 0.01). The age disparity within religions was also significant, with Christian males being 8 years older than Christian females (*p* < 0.01), and Pagan males being 8 years older than Pagan females (*p* < 0.01). Within sexes, Christian females were an average of 2 years older than Pagan females (*p* = 0.03, but no significant difference was found between males across the two religions (Table 5; Figure 4). Specifically, Pagan females were on average 17 years at marriage, while Christian females were on average 19 years at marriage. Overall, the average age of females at marriage was 18 years. Pagan males were on average 25 years at marriage, while Christian males were on average 27 years at marriage. Overall, the average age of males at marriage was 26 years.

**Table 5.** Tukey’s honest significant difference between the ages of marriage for both sex and religion for the Roman model predicting age of marriage.

Religion/sex	Difference	Lower CI	Upper CI	*p*	Significance
Male versus female	7.78	6.65	8.92	0.00	***
Pagan versus Christian	−1.84	−2.92	−0.76	0.00	***
Male: Christian versus female: Christian	7.79	5.74	9.85	0.00	***
Female: Pagan versus female: Christian	−1.89	−3.64	−0.14	0.03	
Male: Pagan versus male: Christian	−1.74	−4.16	0.67	0.25	
Male: Pagan versus female: Pagan	7.94	5.77	10.10	0.00	***

CI: confidence interval.

Significance: ***p < 0.001.

#### Polygyny model

**Figure 5.**
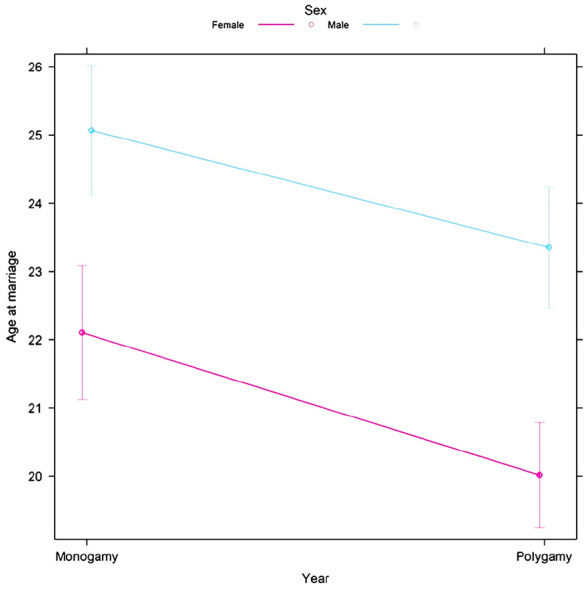
Ages at marriage of males (blue) and females (pink) in monogamous and polygynous relationships in Utah, USA, who were born between 1774 and 1885 as predicted by the Polygyny model. Error bars represent 95% confidence intervals.

Figure 5 was created to visualize the age disparity in polygynous relationships. Overall, at the first marriage, males were significantly older than their female counterparts by 3 years (*p* < 0.01; Figure 5). Specifically, monogamous males were 3 years older than their female partners (*p* < 0.01), while polygynous males were 3 years older than their female counterparts at the time of their first marriage (*p* < 0.01). In polygynous relationships, the age of the second, third, and fourth wives was significantly different at their time of marriage but by a very small amount, namely, 0.67 years (*p* = 0.05), while the age of the husband continually increased by an average of 8 years with each subsequent marriage (see SI: Polygyny model). In general, polygynous marriages were significantly younger than monogamous ones, but only by 0.75 years (*p* = 0.01; Table 6). In comparing between the same sex in different marriage types, neither were significant.

**Table 6.** Tukey’s Significant Honest Difference in means of age of marriage between sex, marriage type, number of wives in the relationship, and order of wives for the polygyny model predicting age of marriage.

Marriage type	Difference	Lower CI	Upper CI	*p*	Significance
Polygyny versus monogamy	−0.75	−1.35	−0.15	0.01	*
Sex	Difference	Lower CI	Upper CI	*p*	Significance
Male versus female	3.28	2.70	3.86	0.00	***
Marriage type + sex	Difference	Lower CI	Upper CI	*p*	Significance
Male: Monogamy versus female: monogamy	2.88	1.57	4.19	0.00	***
Female: polygyny versus female: Monogamy	−0.95	−2.01	0.10	0.09	
Male: polygyny versus male: monogamy	−0.54	−1.77	0.69	0.67	
Male: polygyny versus female: polygyny	3.29	2.34	4.25	0.00	***
Wife order	Difference	Lower CI	Upper CI	*p*	Significance
Second wife versus first wife	1.15	0.24	2.07	0.01	**
Third wife versus first wife	−0.09	−1.66	1.47	1.00	
Fourth wife versus first wife	−1.23	−4.81	2.35	0.81	
Third wife versus second wife	−1.25	−2.96	0.46	0.24	
Fourth wife versus second wife	−2.38	−6.03	1.26	0.33	
Fourth wife versus third wife	−1.14	−4.99	2.72	0.87	
Number of wives	Difference	Lower CI	Upper CI	*p*	Significance
Two wives versus 1 wife	−0.38	−1.23	0.48	0.67	
Three wives versus 1 wife	0.47	−0.60	1.54	0.67	
Four wives versus 1 wife	1.24	−0.35	2.83	0.19	
Three wives versus 2 wives	0.85	−0.16	1.85	0.13	
Four wives versus 2 wives	1.62	0.07	3.17	0.04	
Four wives versus 3 wives	0.77	−0.91	2.45	0.64	

CI: confidence interval.

Significance: *p < 0.05; **p < 0.01; ***p < 0.001.

### Potential correlates

**Figure 6.**
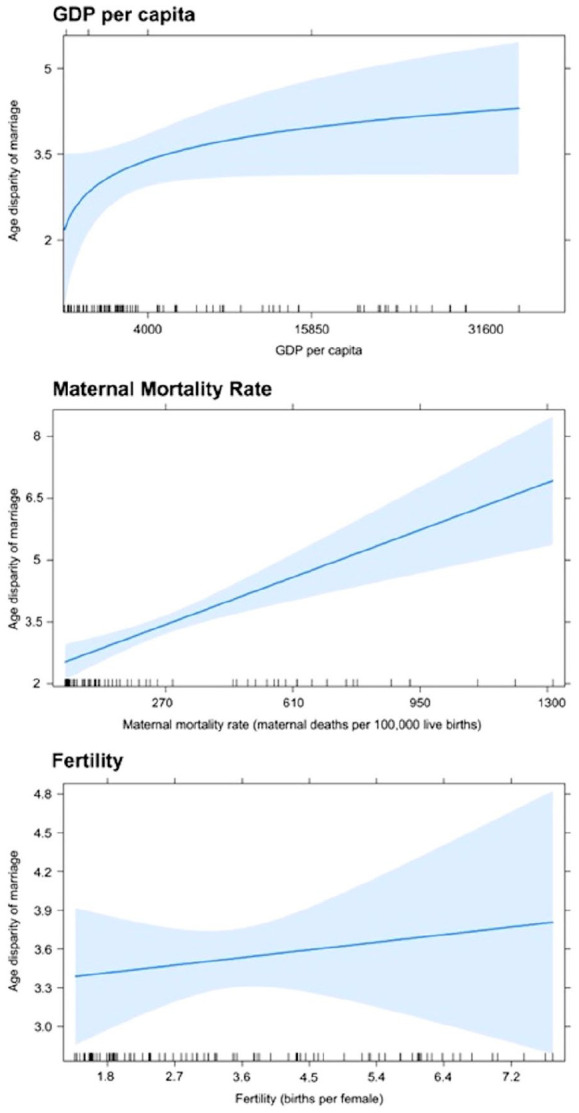
The relationships between age disparity in marriage and gross domestic product per capita, maternal mortality rate (maternal deaths per 100,000 live births), and fertility rates (births per female) between 1990 and 1998 in 99 countries around the world as predicted by the social correlate model. Shaded regions represent 95% confidence intervals.

**Figure 7.**
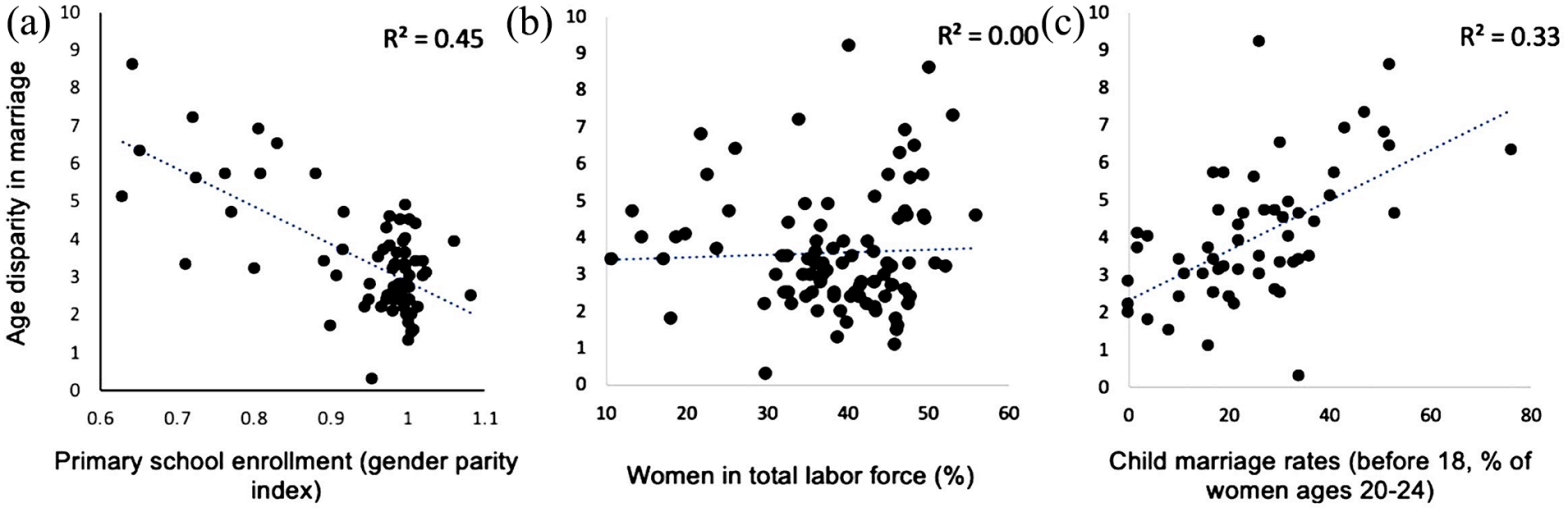
Age disparity in marriage plotted against primary school enrollment (a), women in total labor force (b), and child marriage rates (c). Note: primary school enrollment is measured using a gender parity index, where smaller ratios indicate less females than males enrolled in primary school, and ratios of 1 indicate an equal ratio between females and males.

Figures 6 and 7 explore potential relationships between age disparity in marriage and several social factors. The overall age disparity predicted by our model was 3 years between males and females (Table 7; Figure 6). GDP per capita did not significantly predict age disparity at marriage, but only by a very small margin (*p* = 0.07). MMR was very strongly associated with age disparity, increasing by 275 maternal deaths/100,000 live births for each additional year of age disparity (*p* < 0.01) between partners. Fertility also did not significantly predict the age disparity at marriage (*p* = 0.57).

**Table 7.** Coefficients and significance of parameters of social model predicted relationship between age disparity, maternal mortality rates, fertility, and wealth.

	Estimate	Standard error	*z* value	Pr(>|*z*|)	Significance
Intercept	−0.04	0.08	−0.47	0.64	
Log GDP	0.19	0.10	1.82	0.07	
MMR	0.76	0.17	4.57	0.00	***
Fertility	0.08	0.14	0.56	0.57	

Significance: ***p < 0.001.

**Figure 8.**
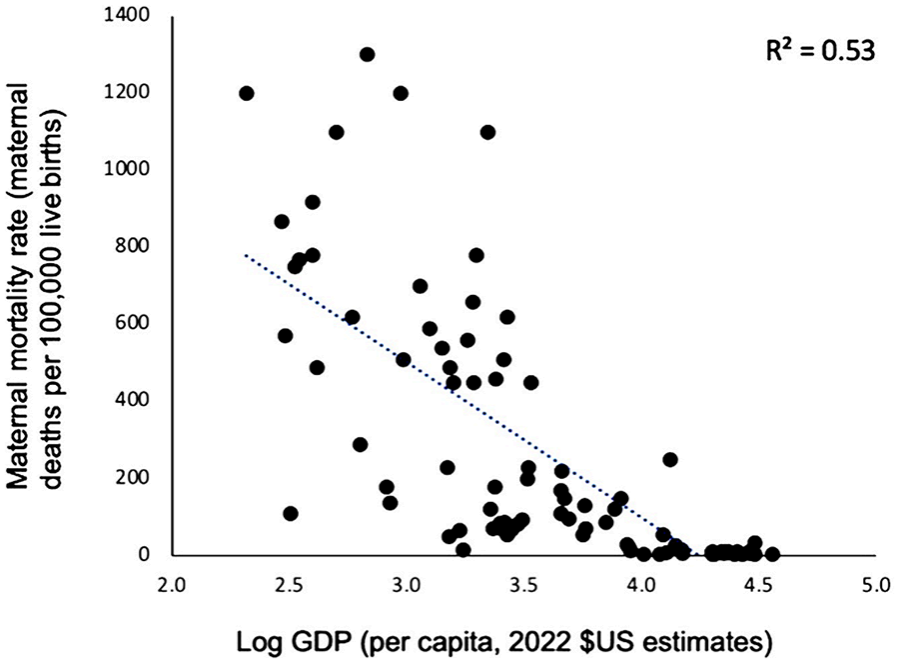
Maternal mortality rates plotted against log gross domestic product (2022 US$ estimates).

When investigating the correlations between age disparity with primary school enrollment, child marriage rates, and women in the labor force, the strongest correlation was found between age disparity and primary school enrollment (*R*^2^ = 0.45, *r* = −0.67). Lower correlations were found between age disparity and child marriage rates (*R*^2^ = 0.33, *r* = 0.57). No correlation was found between age disparity and the percentage of women in the total labor force (*R*^2^ = 0.00, *r* = 0.04). Negative relationships were observed between age disparity and primary school enrollment. Positive relationships were observed between age disparity and child marriage rates. Of the top 15 highest age disparities in marriage in the world, Burkina Faso, Guinea, and Nigeria were also within the top 15 highest rates in maternal mortality, child marriage, and polygyny (for a complete list, see SI).^31,40,45,47^ Regarding Figure 8, the primary intention was to explore the relationship between a nation’s wealth and its MMR. A negative relationship between the two variables was observed (*R*^2^ = 0.53, *r* = −0.73).

## Discussion

The central goal of this study was to evaluate the assumption of the mate choice theory regarding men selecting women younger than themselves as mates. In this study, a positive age disparity (i.e. males being older than females) and a significant difference between the sexes’ age at first marriage were observed among all locations and time frames analyzed (Figures 2–5). From Figures 2 and 3, the general increasing trend across time, more particularly among a female’s age *at* marriage, suggests that historically females were married and/or had childbirth at an earlier age, emphasizing the mate choice theory’s relevancy to ancestral populations and the evolution of maternal mortality and menopause. Figure 4, for instance, demonstrates that Roman females were often adolescents (i.e. between 10 and 18 years) when married; albeit a small data set, it may lend some credibility to the prediction previously described. Overall, Figures 2–5 support the assumption of the mate choice theory that males are, and have been, significantly older than females at marriage. Our results also corroborate other studies which found evidence for the preference for younger females.^48–52^ Interestingly, polygynous males tended to choose females younger and of generally the same age in subsequent marriages, despite being significantly older—further supporting the assumption (Figures 1 and 5). This was also seen in a Brazilian study where they found the tendency for males to choose younger females became more pronounced over their lifespan.^52^ Thus, our results show evidence of the assumption underlying the mate choice theory.

### Age disparity in marriage and the persistence of maternal mortality

In this study, MMR had a strong association with age disparity in marriage, increasing by 275 per 100,000 live births for each additional year in age disparity (Figure 6). To our knowledge, no study has directly explored the relationship between maternal mortality and the age disparity in marriage. However, a few studies have assessed a female’s age at marriage and its relation to MMR. In developing nations, the age of marriage was related to adverse pregnancy outcomes where younger females (i.e. less than 14 years) were at higher risk.^53,54^ In developed nations, however, the relationship was either insignificant or reversed where older women (i.e. greater than 35 years) were at higher risk.^54,55^ With females likely marrying and/or having childbirth at a younger age in the past, as outlined previously, the mate choice theory would propose higher MMR historically.^56^ Hence, although improvements in global MMR trends are generally attributed to health care advancements, they may also be related to increasing trends in women’s age at first marriage or childbirth.

Although a strong relationship between age disparity and MMR was seen, caution must be taken with this finding as there may be contributing or confounding factors. Many areas, for instance, which see high age disparities in marriage also have greater inequities in health care accessibility, wealth, and social norms.^57–59^ Figure 8, in particular, demonstrates a strong correlation between a country’s wealth and its MMR, suggesting the importance of health care resources in reducing maternal mortality. In addition, the arguments proposed by the mate choice theory may not be relevant to current and/or developed societies where females marry at later ages and polygyny are uncommon. Greater access to health care resources further complicates findings in developed regions since it would reduce the incidence of maternal mortality caused by youth-related complications. Ultimately, maternal mortality is complex with multiple contributory factors, one of which is likely the age disparity between couples.

### Age disparity in marriage and the evolution of menopause

Evidence of age–disparate relationships also upholds the assumption underlying the mate choice theory’s relevancy to menopause (Figure 1). Considering that historically females married and had children soon after puberty, the current age disparity likely evolved from a higher point under our more promiscuous/polygynous past.^60,61^ While Morton et al.’s^14^ simulation produced a model representing menopause under the assumptions of male preference for younger women and infertility mutations. Le et al.^62^ noted that any deviations to the preference counteracted the evolution of menopause. Specifically, mentioning that the mate choice theory “needs further explanation of how a male strategy to exclusively mate with young females could have arisen in our common ancestors and remained evolutionarily stable for long enough to drive the evolution of old-age female infertility.”^62^ Since the preference for younger females is a universal phenomenon, as shown in our results to have persisted in the past and among many countries, the evolution of men’s preference for younger women in a universal patriarchal society is not hard to imagine. For the mate choice theory to hold, the preference for younger women need not have started as an exclusive preference to begin with but to have evolved and intensified over time. The persistence of maternal mortality would likely have further contributed to and hastened the evolution of menopause if it forced men to choose even younger, unmarried, and available females, lowering the overall age of fertile women beyond which infertility mutations would begin to accumulate (Figure 1).^13,14^ However, as a new theory, more research must be done to help elucidate our understanding as it is difficult to predict how deviations or other factors, such as delayed reproduction, would affect the system. It would be beneficial to observe the relationship between the average age disparity in marriage and the onset of menopause. This may provide further evidence for the mate choice theory as it predicts that the evolution of delayed marriage/childbearing, which is usually accompanied by a smaller age disparity between couples, would result in a delay in menopause.^14,63^

### Why do age–disparate relationships persist?

The relationship between GDP, maternal mortality, and fertility with the age disparity in marriage was explored in this study (Figure 6). MMR significantly predicted age disparity between couples, and GDP per capita was very close to significant (Figure 6). When independently correlating other social factors with age disparity, strong correlations were not seen, but a moderate correlation was observed between age disparity and primary school enrollment, suggesting that more educated females may choose males of more similar age to them (Figure 7). Several other studies have also found early marriage to be more prevalent among females with less education.^58,59^ This may be due to a number of factors. For one, less educated females may have greater pressure to marry young compared with those who continue to pursue education, imparted by either an external source from family/society or internally driven.^64–66^ These individuals may then prefer or be pressured to choose older men, who have more time to accrue greater financial worth, as they may be unable to work or enter a high-paying job in their society.^67,68^ Males may further exploit this financial vulnerability to choose young females, especially if socially rewarded or given the opportunity.^67,69^ By the same logic, this may also explain the weak–moderate correlation observed between age disparity and child marriage rates. Early marriage can also be the cause of having less education as females may be expected to take on housekeeping or childrearing roles, as seen in many developing countries.^58,70–72^

A complication to the idea that females choose older males due to financial resources lies in the finding that when age disparity and the percentage of women in the total labor force were plotted against each other, no correlation was found (Figure 7). It was predicted that more females in the workforce would correspond to a lower age disparity since females would have less financial need. Although several studies have shown that working women tend to delay marriage, there are limited studies available on how that impacts the age gap in marriage.^73–75^ Perhaps, rather than financial need resulting in greater age disparities in marriage, education may instead instill certain values in females, such as self-efficacy and independence, which reduce their need or desire to marry someone older.^73,74^ Unexpectedly, our model did not demonstrate a significant association between GDP and the age disparity in marriage, indicating that age–disparate relationships are more complex and cultural than resource consideration (Figure 6). This is in contradiction to a study done by Feng and Ren^76^ which found increases in GDP associated with decreases in the age disparity between marriage. All things considered, men’s drive for younger women may still remain an important factor in maternal mortality and menopause, and this drive may have been reinforced by selection due to its correlation between beauty and fertility.^11^

### Research implications

With the mate choice theory founded on the assumption of age–disparate relationships, the results of this study confirmed our hypothesis that males are and have been older than their female spouse in the past. However, it is important to emphasize that our samples may not be generalizable to all locations and time frames. While we attempted to collect data sources diverse in location and time frame, it was particularly difficult to find data before the 1800s and from non-Western cultures. Thus, the results from this study may be more pertinent to Western cultures and more current times. In addition, by no means is this study stereotyping all age–disparate relationships as being harmful to female health and well-being, but rather to shed light on the potential implications of child marriages and large age–disparate relationships to female health. Currently, pregnancy and childbirth are the leading cause of death in women aged 15–19 in developing countries.^77^ Although improvements in health care access to women are an important action to reduce MMR, this study also points to the importance of the age disparity in marriage. Mandating laws against marriage before the completion of secondary education may help reduce the age disparity and thus MMR, but can be a controversial or complicated stance.^70^ A coordinated action will be required and should include aims toward stronger enforcement of child marriage laws, working with community or faith leaders, and making education more accessible to females.^70^

### Limitations and future studies

In this study, age disparity in marriage was used to explore the mate choice theory. However, the crucial factor in studying the relationship between age disparity in marriage and maternal mortality is not the absolute age difference but the age of the female at first birth, especially as some couples may delay pregnancy or not have children. Thus, our data likely reflect ages younger than what is relevant to maternal mortality, which is the age at childbirth. It is important to note, however, that such planned parenting is a recent phenomenon.^78^ Although measuring the age disparity in marriage may not be an accurate measure of the age of women at first birth, it was critical to assess the specific preference for younger females, as assumed by the mate choice theory. Future studies should assess the correlation between the age at marriage and the age of women at first birth. The use of correlations to explore the theory also presents limitations on the validity and generalizability of the results and implications. The strong association between MMR and age disparity, for example, should be approached cautiously. Again, this study is not trying to push that age disparity in marriage is the only or most important factor toward maternal mortality and menopause. The main objective was to assess the assumption of age–disparate relationships in the mate choice theory.

In addition, a few sources of data used in this study may pose challenges to the generalizability of the results. For one, polygynous data were only utilized from a sample of Utah Latter-Day Saints.^33^ Although the results from this source were corroborated by other studies in literature, different results may be found among different polygynous groups. In addition, of the data collected from Ancestry.ca, a large proportion of individuals had English names, even those collected from South African and Indian marriage registries.^25–29^ The collection used for South Africa was taken from registries from the Dutch Reformed Church which predominantly consists of South Africa’s White population.^28^ Thus, the results found may be more representative of that religious denomination. Regarding the Indian sample, Ancestry.ca did not specifically disclose where the marriage records were taken from.^27^ In both cases, it is difficult to predict how these inconsistencies would have affected the data since they could have either inflated or deflated the ages observed. For instance, if these individuals were immigrants of European descent, they may have had a higher SES and be more educated, inflating our results. On the contrary, it may have also deflated our results if their religion values younger childbirth. Ultimately, since we decided to perform a comparative study rather than a global analysis of each country’s age disparity in marriage, it is important to address the implications of analyzing a select number of regions. These areas, consisting of more complete data, likely represent wealthier or more developed regions. Within these locations, the data collected from censuses may only represent citizens who were accessible for collection, overlooking those living in inaccessible or remote areas. In regard to our sample from the UN, we also wanted to note the shortcoming in the inability to account for time-related variation since the data were collected from a cross-section in time.^30^ Ideally, the best data supporting the mate choice theory would track the age disparity in relationships over time, from a diverse set of locations.

## Conclusion

Overall, the results from this study confirm the assumption underlying the mate choice theory since all locations and time frames analyzed had a positive age disparity in marriage. The strong association between MMR and the age disparity in marriage may also lend strong support for the theory’s assertions on maternal mortality. Thus, maternal mortality may be addressed by aiming to reduce the age difference between couples, specifically through child marriages. However, these issues are complex with multiple contributing factors. Primary school enrollment, for example, may be of importance, given its stronger correlation observed with age disparity in marriage. The prevalence of age–disparate relationships may also support the theory’s arguments concerning menopause. With the general trend of decreasing age disparity between couples, it is predicted that the age of perimenopause onset may get pushed forward. Future studies should seek to assess the correlation between age at marriage and age of women at first birth and track the age disparity in couples over time, from a diversity of locations. Through its effect on maternal mortality and menopause, the age disparity in marriage has likely been an important factor affecting women’s health.

## Supplemental Material

sj-docx-1-whe-10.1177_17455057241264687 – Supplemental material for Cost of mate choice: Changing patterns of global age disparity in marriage and their consequences to women’s health including maternal mortality and menopause Supplemental material, sj-docx-1-whe-10.1177_17455057241264687 for Cost of mate choice: Changing patterns of global age disparity in marriage and their consequences to women’s health including maternal mortality and menopause by Mindy Pru, C Michelle Brown and Rama S Singh in Women’s Health

## Declarations

**Ethics approval and consent to participate:** Ethics approval was not required as this study was based on data collected from literature.

**Consent to participate:** Not applicable.

**Consent for publication:** Not applicable.

**Availability of data and material:** Not applicable.

**Code availability:** Not applicable.
